# Designing a range of mentalizing interventions for young people using a clinical staging approach to borderline pathology

**DOI:** 10.1186/s40479-020-0121-4

**Published:** 2020-03-12

**Authors:** Joost Hutsebaut, Martin Debbané, Carla Sharp

**Affiliations:** 1 Viersprong Institute for Studies on Personality Disorders, Halsteren, the Netherlands; 2 Centre of Expertise on Personality Disorders, Utrecht, the Netherlands; 3 Developmental Clinical Psychology Research Unit, Faculty of Psychology and Educational Sciences, University of Geneva, Geneva, Switzerland; 4 Research Department of Clinical, Educational and Health Psychology, University College London, London, UK; 5 Department of Psychology, University of Houston, Houston, Texas USA; 6 University of the Free State, Bloemfontein, South Africa

**Keywords:** Early intervention, Borderline personality disorder, Mentalization-based treatment, Clinical staging

## Abstract

Borderline personality disorder (BPD) can have a long-lasting impact on social and professional functioning, even when core symptoms of BPD are in remission. Adolescence may be a critical developmental period to change the potential long-term functional outcome of BPD. This paper presents a range of mentalizing interventions to alter the course and outcome of BPD, based upon a model of clinical staging. Mentalizing interventions have in common a focus on strengthening self-regulatory and interpersonal capacities, aiming to improve adaptive social learning. This paper argues that these interventions should be dosed and organized according to the stage of progression of BPD, which is illustrated by discussing different specific formats for mentalization-based interventions, including an early-intervention program for BPD and a standard program for full BPD.

## Background

Borderline personality disorder (BPD) is a severe mental condition associated with an increased risk of traditional axis I disorders, reduced life expectancy and severely impaired social and occupational functioning [20, 26, 36]. Longitudinal data draw a picture of a potentially chronic condition characterized by patterns of remission and relapse at the symptom level, as well as a more pervasive and persistent disability in areas of work and romantic relationships [56, 58]. Maladaptive self-regulation and interpersonal function have been identified as the common core of personality pathologies [1, 48]. Although most outcome studies adopt remission of BPD diagnosis and symptoms as a primary outcome, from a patient perspective, social and professional recovery may be more important. Therefore, one of the main challenges in treating BPD may be the design of interventions to prevent or recover from social and professional disability and help patients to establish a meaningful life, or as Linehan [35] referred to “a life worth living”. Notably, this challenge may be similar in other areas of severe psychopathology [2]. Meta-analyses in the field of psychosis have demonstrated positive outcomes with regard to psychotic symptoms and associated problems, but much less favorable outcomes for social functioning, reflecting a similar symptom-disability gap [7].

In order to prevent or change the unfavorable functional outcomes of BPD, it may be helpful to switch the traditional focus from treating BPD in adulthood to addressing emerging BPD in adolescence [11]. Although the underlying impairments in self and interpersonal regulation may have their origins early in life, typical BPD symptoms usually have their onset in puberty [29]. Despite resistance against the diagnosis before 18 [33], there is nowadays accumulating evidence that a PD diagnosis is prevalent in young people (e.g., [27, 30, 59]), distinguishes reliably between normative and clinical development [5, 15, 55, 56] and predicts a whole range of current and future negative outcomes, including conduct and school problems [31, 53, 33] substance abuse [42], emergency admissions [32] and deviant sexual behavior [38, 51].

Interestingly, there may be a different timeframe for the development and/or expression of several types of BPD symptoms. Typically, hormonally and neurobiologically driven symptoms of affect and impulse dysregulation will emerge early in the course of the disorder, with typical first clinical expressions of mood instability, anger problems or risky behavior [52]. These symptoms therefore may provide sensitive markers for emerging BPD. Symptoms related to interpersonal and social functioning may often emerge only by the end of puberty [14], possibly because of the increasing demand on autonomy and the associated need to relate to and learn from peers. Developmental issues require adolescents to engage with peers and adolescents lacking basic abilities in social cognition and relatedness, may become socially isolated and devoid of opportunities to learn socially from healthy peers. This may contribute to an increasing gap between the course of personality development in typical adolescents and adolescents with BPD. While typically developing adolescents are able to overcome their developmentally induced storm and stress, adolescents with BPD seem to become socially more disabled, contributing to a more persisting clinical picture [16]. In line with these data, it has been demonstrated that the inter- and intrapersonal symptoms of BPD indeed provide better discrimination as adolescence age into early adulthood [46]. Therefore, it may be hypothesized that the severity of impairments in social cognition, social learning and connectedness – while not being always clearly prevalent in the early manifestations of the disorder – may underpin the potential progression of BPD towards a more chronically impairing disorder.

Adolescence is not only the developmental phase during which BPD symptoms become prevalent and therefore potential indications for treatment, it also plays a crucial role in the course of BPD as the foundations for adaptive social and professional functioning in (young) adulthood are being established in adolescence [43]. Success at school and ability to socially bond with peers may pave the way towards social and occupational functioning in adulthood. Young persons who fail to complete these developmental tasks in adolescence may be severely handicapped to design an adult life that includes full social participation. Adolescent BPD has been demonstrated to be associated with increased risk for academic underachievement and school dropout [31]. Failing to complete school and to establish social bonds with peers in adolescence may predispose to the social and occupational disabilities of adult BPD patients. Developmentally, it may even be argued that the developmental break-down of (some) BPD patients, resulting in social isolation and professional withdrawing, may reflect a failure to cope with the complexities of social life. While this may on the one hand help to dial down the heftiness of BPD symptoms by avoiding socially threatening contexts, it may ultimately result in a far-reaching disability in social and occupational function. As a consequence, adolescence may be a critical developmental period to change the course of BPD, particularly regarding its potential chronic impact on *functioning*. Preventing developmental break-down in adolescence by keeping young people in school and scaffolding healthy relationships with peers may therefore be the most effective strategy to prevent the long-term impact of BPD on future social and professional functioning. This may include improving peer relationships and overcoming isolation, as incapacity to relate to peers may underpin not only social problems, but also school dropout given the inevitable social nature of educational settings.

To understand and impede the far-reaching impact of BPD – including not only typical BPD symptoms, but also social and occupational disability – a focus on social cognition and mentalizing may be helpful. The concept of mentalizing refers to “a form of imaginative mental activity, namely, perceiving and interpreting human behavior in terms of intentional mental states (e.g. needs, desires, feelings, goals, purposes, and reasons)” [22] with the aim of making sense of ourselves, others, and our relationships. Impaired mentalizing may be a crucial factor mediating the potential deviation from a healthy developmental pathway in adolescents prone to BPD [8]. Conversely, optimal mentalizing may function as an important protective factor scaffolding a healthy transition from childhood into adulthood. Mentalizing fosters social cognition: understanding others and oneself in relation to others may be necessary to complete developmental tasks in adolescence and to engage safely in the social network in school. Critically, neuroscientific research shows the cerebral circuits sustaining mentalizing mature during adolescents [28, 37], and in parallel, experimental studies also suggest improvements in mentalizing during adolescence [17, 39].

There is preliminary evidence that mentalizing interventions improve symptoms of borderline and associated conditions during adolescence [34, 40, 4]. Additionally, evidence from clinical studies suggests that borderline symptoms in adolescents improve with improved mentalizing abilities, suggesting improved mentalizing serves as a mediating mechanism for reducing borderline symptoms [40, 45]. However, it should be acknowledged that results are mixed at best. The MBT group-treatment in the Beck et al. study failed to show superiority above individual psychotherapy sessions. Moreover, only 29% of the MBT-group remitted and 29% attended less than half of the group sessions (Beck et al., 2019). The combined individual and family MBT sessions in the Rossouw and Fonagy (2012) study demonstrated superiority over a heterogeneous control group, but half of the youngsters did not complete treatment and 25% dropped out within the first 2 months of treatment. Both findings – difficulty to demonstrate superiority and high levels of drop out – have also been found in other studies of BPD adolescents [10]. They seem to suggest that the treatment packages offered in these studies may not be appropriate enough to meet the needs of all BPD youngsters involved and warrant for a further refinement.

This paper offers a conceptual framework for designing mentalizing interventions, making two basic arguments. First, we will argue that improving social cognition by improving mentalizing may not only prevent borderline development but may also help to prevent the social disability often associated with BPD, reducing the so-called symptom-disability gap. This paper describes a view on mentalizing interventions to scaffold normative social cognitive development, thereby improving personality functioning in adolescence. The aim of mentalizing interventions may not only be to prevent borderline features to progress into fully established BPD, but also to improve social cognition and thereby improve functioning more explicitly. Second, we claim that treatment effectiveness for young people with BPD in general may benefit from a staging approach. Following other stage-based conceptualizations for prevention and intervention with BPD [9], a core assumption in this view is that the focus, scope and duration of the intervention should be tailored to the stage of progression of BPD. This implies that earlier stages should be targeted with less specialist and less intensive interventions, while later stages should be targeted with more complex, intensive and long during interventions. In other words, selection of interventions is based on a heuristic model of clinical staging [29].

## Staging BPD

Clinical staging is a heuristic strategy to describe the stage of progress of a disease according to its duration and extent. Clinical staging allows a refinement of diagnosis and presents a more dimensional and dynamic approach to assessment of diseases [41]. Two staging models have been presented for BPD [9, 29]. Hutsebaut et al. [29] argue that BPD features represent the generic markers of (severely) impaired personality development. These features are seen as the outcome of partly innate, partly developmentally determined dysregulations in self and interpersonal functioning, which may develop from early on and be expressed in poor attention and affect regulation, impaired impulse control, insecure attachment style or poor regulation of frustrations. Studies on early precursors of BPD indicate that a whole range of childhood disorders related to externalizing (i.c. ADHD, conduct disorders) and internalizing (i.c. mood and anxiety disorders) may precede the BPD symptoms [49]. These child-like expressions seem to lay the foundations for the more specific PD-like symptoms emerging in adolescence [44, 50]. In their view, presented in Table 1, staging BPD implies a structural vulnerability in self and interpersonal regulation (stage 0) with subsequent manifestation of typical symptoms of mood swings, lack of self-confidence and changing self-representations, intense and insecure relationships from puberty on (stages I and II). Studies have suggested that patterns of mood swings and self-injurious behavior typically start to emerge around puberty, while BPD symptoms of avoiding abandonment and intense relationships usually emerge somewhat later in adolescence [13]. Moreover, whereas normative expressions of impulsivity, affective instability and identity disturbance diminish by the end of puberty in normative development, these symptoms seem to stabilize or increase in BPD adolescents, creating a larger gap between normative and clinical development by the end of puberty [15]. Chronic impairments in self and interpersonal regulation often become associated with chronic or returning patterns of these BPD symptoms, associated with high levels of ‘co-morbid’ psychopathology and pervasive social and professional disability (stage III). Indeed, Zanarini’s landmark follow-up study shows periods of remission and relapse in BPD symptoms and professional functioning in a significant subsample of BPD patients [58]. These BPD adults seem to go through relatively symptom-free episodes with partial or full recovery, but seem to remain vulnerable for subsequent relapse, with sustained need for treatment [6]. Finally, large subsamples of BPD patients seem unable to recover, leading to persistent disability and often very severe associated psychopathology (stage IV). Although most BPD patients in Zanarini’s sample had periods of symptomatic remission, a group as large as 40% suffered from pervasive and irreversible social and professional disability [58]. New in this staging model is the inclusion of associated psychopathology and areas of disability to determine the stage of BPD progression, extending the scope of BPD stage beyond mere symptoms of BPD.

**Table 1:** Staging model for BPD (adapted from [29])

Stage	Borderline features	Co-morbidity	Social and occupational functioning
Stage 0	No classic symptoms of BPD, but latent impairments in self and interpersonal functioning, expressed in problems in mood regulation, attention deficits, frustration and distress tolerance	Either no formal disorders or some areas of mental problems, including ADHD, conduct problems	No extensive problems, but areas of problems, including school functioning or peer contacts
Stage I	Emerging symptoms of BPD, usually in the areas of affect dysregulation and impulse control	Usually ‘co-morbid’ disorders, including mood, anxiety and conduct disorders	Emerging significant problems in school, peer contacts or relationship between parents and child
Stage II	First episode of full BPD	Usually co-morbid disorders, often in associated areas of emotion dysregulation (mood disorders, PTSD, substance abuse)	Significant and lasting problems in school, peer contacts and family
Stage III	Relapse in full BPD or chronic patterns of full BPD	Usually chronic and multiple co-morbid disorders	Usually recurring significant problems in social and occupational functioning
Stage IV	Full BPD without remission of main problem areas	Usually severe and chronic associated psychopathology	No social or occupational recovery

A staging model is a heuristic strategy that assists in treatment planning [41]. The assumption behind it is that the more BPD has affected different areas of mental, social, academic and other functioning, the more BPD has progressed, implying different interventions may be required. Staging embodies the idea that personality impairment, which is expressed typically by BPD features, is *potentially* progressive and may eventually affect more areas of mental and social functioning. Therefore, interventions should be differentiated according to the stage of progression of personality impairment.

## Common features of mentalizing interventions designed for young people

Mentalizing refers to the capacity to understand (social) behavior by observing and interpreting (accurately enough) the mental states and processes that underpin this behavior [3]. Improving mentalizing in young people enhances their capacity to reflect upon the meaning of behavior, by attending to and trying to make sense of feelings, needs, expectations, and intentions that ‘direct’ this behavior. Mentalizing (well enough) within a social encounter creates a feeling of security and safeness, a feeling of understanding between both parties and a willingness to open up for social learning [21]. Mentalizing creates a state of *epistemic trust*, referring to the openness to learn from others; thus, an individual’s willingness to consider knowledge from someone as trustworthy, generalizable and relevant to the self [23]. A stable mentalizing environment will ultimately scaffold a well-balanced disposition to trust reliable others and still remain sufficiently vigilant towards unreliable others [24, 25].

This paper discusses a range of mentalizing interventions, different in modalities, duration and intensity to fit the needs of each stage of BPD progression. However, these interventions have some features in common which will be discussed briefly. First, mentalizing interventions are less directed at shaping and changing behavior than on strengthening self-regulating capacities and interpersonal abilities. All interventions ultimately focus on improving personality functioning, or to put it in DSM-5 terms: to improve Criterion A function as described in the Alternative Model of Personality Disorders [1, 48]. Criterion A may be understood as representing the ‘building blocks’ of healthy personality development. Mental resilience will be fostered when (young) people learn to develop a sense of self, regulate their emotions, understand themselves, design goals and values in their lives, and when they are capable of establishing safe, rewarding, positive and stable connections with other people. Mentalizing interventions help to strengthen regulatory abilities in circumstances of complex and overwhelming emotions. Second, mentalizing interventions focus on self *and* other. They not only address emotions, needs, and other mental processes in the person, but also in others, in order to better understand other peoples’ intentions and thus select reliable learning opportunities. Mentalizing interventions explicitly address the minds of others as a way to learn to identify reliable minds and prevent misinterpretations of intentions. Third, mentalizing interventions explicitly focus on establishing and using the (therapist) relationship as a vehicle to unlock social learning [23]. They may explicitly address what is happening between the therapist (counselor, teacher, parent) and patient (young person) and use this relationship as a mirror of other relationships enabling learning through experience and establishing renewed opportunities for learning. Establishing epistemic trust within the therapist encounter is considered to be an essential part of any intervention, but also requires a focus on potential ruptures that close down social learning within treatment. Finally, all interventions focus on the context of the individual. Learning from others not only requires detecting favorable learning opportunities, but also an engaged and reliable environment. Opening up for detrimental learning environments may be iatrogenic. For young people, including context usually implies involving families, but could also imply the inclusion of peers, teachers or other key persons in the lives of these youngsters.

## Mentalizing interventions for different (early) stages of BPD

Borderline impairment is dimensional and potentially progressive, as is all personality pathology impairment. This requires interventions that are tailored to the stage of borderline impairment. Early intervention for young people with BPD will typically involve interventions at stage 0, I-II, and II-III, referring to targeting ‘borderline disposition’, early stages of BPD, and severe and already chronic stages of BPD. Table 2 gives an overview of these different interventions.

**Table 2:** Overview of characteristics of mentalizing interventions

	Kids@risk	MBT-early	MBT-A
For whom?	Stage 0, 12–13 years	Stage I-II, on average 13–15 years	Stage II-III, on average 15–17 years
Structure	Low dosage: Single sessions, courses	Medium dosage: 16 weeks + 6 months booster	High dosage: 4 months pre-treatment, 9–12 months intensive treatment, 3–6 months post-treatment
Modalities	Single modality: Psycho-education, class sessions	Multi-modality, with accent on individual therapy, but including family sessions, case management, and occasionally medication review	Multi-modality: Group therapy, individual therapy, family therapy, case management, medication review
Team	Trainer, teacher	Individual therapist, team-based	Multidisciplinary team

### Mentalizing interventions for stage 0(−I)

These interventions will typically have a broad scope and involve children and young people with a clear disposition for psychopathology as well as those who are not necessarily on track to develop psychopathology. Interventions can 1) be community-based, school-based, or otherwise closely related to general community services and not be restricted to mental health care; 2) be focused at creating a mentalizing environment at home, school and in youth communities; 3) create sensitivity of parents, teachers and other adults to detect young people at risk.

Interventions at stage 0 may be psycho-educational in nature and assist parents and teachers to take a mentalizing stance and support personality development in children and young people. The Dutch Kids@risk project focuses on parents, teachers and young people of 12–13 years (www.kidsatrisk.nl). It provides psycho-education for teachers of the first grade of secondary school and for parents of young people. The focus is on helping teachers and parents to take a curious and reflective stance towards young people and reflect upon their behavior. Instead of ‘labelling’ behavior (ADHD, aggression problems, difficult home situation), teachers are stimulated through a series of exercises to take a different, complementary reflective stances to trigger reflectivity (e.g. wondering why the pupil is displaying a particular behavior at a certain moment and what trigger the teacher may have provided for this behavior, examining this from different points of view). In the same project, similar information and exercises are offered to parents. The aim is to help these key persons to be more aware of the mental processes in themselves and in the young people and to support them to better understand their pupil’s behavior. Moreover, by reflecting upon the class atmosphere and the interactions among pupils, teachers are helped to create a more supportive, safer environment in the class, in which emotional contents may be discussed. In addition to the interventions offered to teachers and parents, the project also includes class sessions aimed at discussing basic themes like emotions and self-image among young people. Similar more preventative mentalizing interventions have been piloted in parents and children or community-based caregivers and children.

### Mentalizing interventions at stage I(−II)

MBT-early is an early-intervention program for young people with emerging BPD. It was designed for youngsters in an early stage of BPD, including youngsters with (subclinical) borderline features or full BPD, limited in time and with (rather) limited associated psychopathology and/or developmental break-down. Typically, young people will be 13–15 years, have 3–5 features of BPD, may have additional mood or conduct problems/disorders, but will often have some relatively intact areas of functioning, like at school or at home. MBT-early integrates the principles of Helping Young People Early (HYPE, [11]) with a mentalizing treatment approach. The program is based upon the following principles: 1) systematic screening for borderline features to detect incipient cases of (still subclinical) BPD; 2) rapid response (avoiding waiting lists); 3) offering an all-in-one package, including individual and family treatment, case management, crisis management, and medication review; 4) fostering resilience through a focus on (improving mentalizing with regard to) developmental tasks, including school, and peer and parent relationships, instead of (merely) focusing on symptom improvement; 5) using an empowering stance and offering intermittent episodes of care instead of long treatment trajectories. The primary aim of MBT-early is to scaffold personality functioning and development, in the hope of preventing BPD symptom patterns from becoming chronic and to circumvent developmental arrest.

MBT-early has a two-stage treatment, similar to HYPE-CAT [10]. It starts with an active treatment phase, lasting for 16 weeks, with a weekly session. The first 3–4 sessions are used to make the joint formulation and collaborate on treatment goals. The next ten sessions are used to focus on two individually determined treatment goals, usually one rather symptom-oriented goal (e.g. reducing suicidal thoughts, reducing aggressive outbursts, improving mood swings) and one rather developmentally focused goal (improving peer relationships, enhancing self-competency). The last two sessions are used to formulate a relapse prevention plan and to write and discuss a review letter. Additionally, 3–4 family sessions are scheduled, besides case management (teacher contact, …) and two review sessions with the family. The active phase is followed by a booster phase, with scheduled contacts after 1, 2, 4 and 6 months and provisional crisis contacts. After this booster period, the file may be closed, or an additional treatment episode may be initiated (e.g. 4–8 sessions). The young person may contact the treatment team also after closing the file. If necessary, an additional episode of treatment may be started, reflecting the idea of intermittent treatment.

The active phase of treatment is focused on establishing a mentalizing process in the young person and his environment. The goals of the young person are mainly used to motivate him/her to engage in a mentalizing process, by exploring mental states and processes related to (changes in) goals, e.g. by exploring precursors and mental processes associated with changes in mood, or with problems in peer relationships. The aim of the booster period is to empower the young person and support this initiated process of improved mentalizing as a way to improve general functioning. By allowing youngsters to contact the team also after closing the file, MBT-early aims to prevent iatrogenic damage when former symptoms re-emerge. The general stance in MBT-early is focused on empowerment and increasing self-competence.

All individual therapists in an MBT-early team conduct all interventions, except for the medication review. They conduct individual and family sessions, attend school visits, and schedule treatment review sessions. All are trained at least at the level of basic MBT-therapist, including a 3-day training and 8 supervisory sessions in MBT. Individual therapists come together in team meetings, reviewing each case briefly every week, and more extensively every month.

### Mentalizing interventions for stage II (−III)

MBT-A is an intensive, multi-modal and extended intervention assigned to young people with (already) severe and chronic patterns of BPD symptoms, high levels of associated psychopathology, and significant problems in social and school areas. Youngsters are typically 15–17 years, have full-blown BPD and at least 1 or more comorbid symptom disorders, including substance abuse disorders, eating disorders and PTSD. There are often significant problems in more than one area of life, including high absence or dropout from school, outplacement from home and/or engagement in negative peer groups, as well as self-harm and suicidal behaviors. These youngsters are at high risk for chronic BPD beyond adolescence, including the range of mental and social disabilities as discussed earlier. MBT-A is grounded in the evidence based adult MBT-programs with age-specific adaptations. It includes different treatment modalities performed by a multidisciplinary team. MBT-A is organized in three stages. It starts with a pre-treatment phase, including individual and family assessment, medication review, crisis planning, formulation of treatment goals (individual and family), MBT-introductory course (a psycho-educational course for youngsters and their parents) and school and home visits. After 3–6 months, when individual and family commitment is established and crisis is managed sufficiently, young people start the intensive phase of treatment. This includes weekly individual treatment, weekly group treatment, weekly case management and crisis planning, and bi-weekly family sessions. The intensive phase usually lasts 9–12 months and is followed by a post-treatment maintenance period of 3–6 months, usually containing individual and family sessions. Treatment is offered by a multidisciplinary team, including individual psychotherapist, individual nurse, group therapist, family therapist and psychiatrist. All team members are trained at least at basic level in MBT and the principal therapist is trained at MBT-therapist level.

MBT-A has a more comprehensive focus as compared to MBT-early. It follows the structure of MBT, combining different modalities of therapy (individual, family, group) and following five overarching goals: commitment to treatment, reducing symptoms of co-morbid disorders, improving interpersonal relationships, reducing self-destructive behavior, and improving social and occupational functioning. Additionally, similar goals are formulated for the MBT-F based family therapy: commitment, improving mutual relationships, reducing family crisis, and improving parental competence. In order to improve consistency, the team meets regularly besides formal review sessions, to discuss patients and families.

## Clinical implications: two vignettes to illustrate a staging approach to borderline pathology

To demonstrate how a staging approach to borderline pathology may inform treatment assignment, we will briefly discuss two cases representing different stages.

### Case 1

Charlotte is a 14-year old girl, referred for treatment after a medication overdose. She has been feeling depressed for a while, having suicidal ideations regularly. She comes from a broken family, lives with her mother and has a disturbed relationship with her father. Her depressive moods started about 2 years ago, after having started secondary school. Her mother recounts she had always been an anxiously attached girl, and she claims her daughter feels abandoned by her father who would only be irregularly available to her. Charlotte started to display self-injurious behavior half a year ago, after experiencing some issues with other girls from school. Diagnostic assessment reveals a persistent depressive disorder, but also four BPD features: affective instability, suicidal ideation and self-injurious behavior, identity disturbance and impulsivity, as reflected in risk taking behavior and occasional binge eating. However, she attends school and has social contacts with peers inside and outside school, although conflicted at times. She has an intense, but ambivalent contact with her mother, who feels extremely worried, especially since the overdose, reflected in overcontrolling parenting (e.g. checking her being okay every 15 min when she’s in her room).

### Case 2

Lucy is a 16-year old girl, referred by a psychiatrist from a general mental health care center, where she had been in treatment for a depressive disorder. She suffers from mood problems and – as she calls it herself – ‘social anxiety’. She feels easily judged, fears being abandoned and consequently withdraws from social contacts. She mentions a pervasive distrust towards others and doesn’t open her mind to anyone, to ‘protect herself from being hurt’. She cuts and burns herself, is chronically suicidal and admits to spare medication ‘in case I need an escape’. Occasionally she has extremely intense relationships with friends, but fails to keep longstanding friendships. Her parents feel they have lost their daughter for several years. They were informed about her suicidal and parasuicidal behavior through a teacher from school she trusted more than her own parents. They feel they have no access to their daughter and disagree among each other on how to deal with her often irritable behavior at home. They’ve had family therapy together, but felt it damaged their relationships more, given the extreme arousal it created in all the family members. Lucy met criteria for several mental state disorders, including recurring depressive disorder, social anxiety disorder, eating disorder not otherwise specified and some features of PTSD, reactive to a sexually abusive incident she had with a boyfriend. Additionally, she meets criteria of BPD and several features of Avoidant PD. She meets BPD criteria for: frantic efforts to avoid abandonment, intense relationships, identity disturbance, impulsivity, (para) suicidal behavior affective instability, emptiness and dissociation under stress. She still attends school, but on irregular basis, agreed upon after a consult between school and her psychiatrist. She feels socially isolated, although she enjoys playing theatre, which she calls ‘the only place where she can be herself’.

Both girls were screened at admission for features of borderline pathology, using the McLean BPD screener [60]. They were administered the SCID-I [18] and SCID-II [19]. Additionally, a clinical interview was conducted to design a picture of their social functioning at school, at home and with peers. Although both youngsters clearly display features of BPD, there are also important differences, suggesting a different stage of the disorder, with Charlotte showing (still) more intact functioning in different areas and less pervasive psychopathology than Lucy. Matched with the staging model (Table 1), Charlotte seems to meet the stage I profile (although probably progressing to stage II), while Lucy matches the stage II profile (although also probably already progressing to stage III). Their different stage profiles would entail different treatment needs, with Charlotte still being eligible for an early intervention approach, i.e. MBT-early, while Lucy’s complex picture of psychopathology and developmental arrest, may warrant a more intensive and comprehensive treatment approach, i.e. MBT-A. Implicated in this staging approach is the assumption that although both are teenagers, with significant borderline pathology, their treatment needs may be different, given the clinical stage of borderline progression, with Lucy’s borderline pathology (in the broad sense) being more progressed than Charlotte’s.

## Organization of mentalizing interventions in clinical practice

Approaching BPD from a dimensional staging model means there is a thin line between normative adolescent development and borderline pathology. This should be reflected in the service organization, but more broadly, should ultimately be informed by research specifically examining points of demarcation where this thin line can be more clearly substantiated. Clinically, and following the HYPE approach, the threshold for entering the service should be kept low [11]. Parents and youngsters should have easy access to the service (e.g. facilitating referral, presenting accessible information, providing telephone or chat opportunities). Rapid response to referrals is required, in line with the perspective that early help can prevent unfavorable outcomes. Moreover, integration of community services and mental health care is advised. One way is the creation of networks, including schools, first-line youth services and specialist mental health care centers. These networks incorporate the specialist expertise and make it available for first line services. This may facilitate the detection of youngsters at high risk in a very early stage and prevent iatrogenic first-line strategies during a critical period in very vulnerable youngsters. Furthermore, youth mental health care centers could screen systematically for features of BPD. The rationale is that these features mark high levels of personality impairment and subsequent risk for unfavorable outcomes in different areas beyond adolescence. This requires a change of professional attitude towards the early detection of BPD [12]. Targeting treatment to the underlying impairment in personality functioning (Criterion A) even in the presence of ‘co-morbid’ full diagnoses may enhance resilience and prevent primary and comorbid symptom change or relapse. Assignment for treatment could be based upon a staging model, including the ‘extent’ of ‘co-morbid’ psychopathology and disability, and not merely on a categorical diagnosis of BPD or age. Severely entrenched disorders often need more (intensive and long) treatment than emerging disorders. The more complex and progressed the disorder, the more teamwork may be needed to prevent iatrogenic processes and team problems. This includes increased peer supervision, increased consultation, increased reflective intervision, and more quality monitoring. Reflection among co-workers should be at the center of working with these young people, as their emotional dysregulation and interpersonal sensitivity may also impact upon therapists and counselors. Finally, treatment should be followed-up by empowered self-management of the vulnerability by the youngster and his/her family. Borderline impairment is not ‘cured’ after treatment and adolescent life events may re-install problems. This requires a come-back guarantee, allowing youngsters and families to re-enter treatment rapidly after re-emergence of problems. Intermittent treatment may be an ideal format to balance an empowering stance and a continuing disposition to psychopathology in these youngsters and families [11].

## Conclusion

Borderline personality disorder is not a single disorder [47]. Its defining symptoms rather mark impairments in personality functioning that predispose to a range of symptom disorders and unfavorable outcomes in many areas of life. These impairments tend to become chronic and preclude full participation to social and occupational life. This is probably the worst outcome of BPD: people often fail to fully participate in occupation and social life (Freud’s “love and work”).

This paper argues for the importance of detecting youngsters at-risk early in the course of their problems. BPD symptoms signal a group of young people at risk for developing unfavorable social and occupational outcomes, which – once established – seem to be far more difficult to treat than the core BPD symptoms. Early detection is warranted as the social-cognitive tools for self-regulation, autonomy and responsibility, and interpersonal functioning are sculpted during adolescence and may underpin the potential chronicity of the disorder and its invalidating ‘side-effects’. We argued for a coherent range of mentalizing interventions to address these impairments and support a healthy development. Essential to this perspective is the assumption that this high-risk BPD group consists of young people at different levels of severity and in different stages of the disorder. In our opinion, this heterogeneity may partly explain the observed problems with dropouts and limited general effectiveness in BPD trials in young people, as mentioned earlier (see intro). Given the inclusion of youngsters along the whole range of BPD pathology in these trials, often starting from 2 or 3 BPD features, it seems plausible that these samples consist of youngsters in an early stage as well as youngsters in a more progressed stage of BPD. One treatment package may not serve all young people and the packages offered in these studies – often less intensive and lengthy than in adult treatment studies – may be insufficient for youngsters in a more progressed stage of BPD. One way to proceed and design more effective treatments, may be to refine and personalize treatment packages. Staging BPD could serve as a heuristic strategy to guide this treatment assignment, with more progressed BPD youngsters requiring more intensive and longer treatment. This paper discussed some potential formats for such differential interventions. However, it may be clear that other formats may be possible, following a similar set of principles.

In this paper we also argued that mentalizing interventions may have an additional value to overcoming the symptom-disability gap. Mentalizing focuses on mental states and their value for understanding self and others, enabling young people to learn about themselves and others. The explicit focus within mentalizing interventions on social cognition and interpersonal functioning, may help young people to function more adaptively in social contexts, like class rooms. This may in turn support their capacity to commit to school, which may lay a necessary foundation for occupational participation in adulthood.

This paper is limited in so far that it does not present empirical data to support many of its assumptions. There is no empirical evidence in the field of BPD that early intervention is more effective than late intervention or reduces general costs due to increased resilience. There is also no evidence that progressed BPD stages need different treatment dosages and/or intensity and indeed some studies seem to show that limited amounts of treatment may be beneficial for progressed stages too [57]. Finally, there is no evidence that mentalizing interventions indeed improve functioning more than other PD interventions. Clearly, these are some areas of research that may be important for future studies.

The PD field is still relatively young and has only just began with establishing a more positive and hopeful perspective on the changeability of PDs. A next step may be to personalize treatment approaches, especially in order to improve social functioning. We argued that a staging approach to assessment may improve our treatments by refocusing on early detection and intervention and refining formats of treatment according to the stage of BPD progression. A staging approach may guide future studies, trying to identify ‘what works for whom’.

## Data Availability

NA

## References

[1] American Psychiatric Association (2013). Diagnostic and statistical manual of mental disorders.

[2] Armando M, Hutsebaut J, Debbané M. A mentalization-informed staging approach to clinical high risk for psychosis. Front Psychiatry. 2019. 10.3389/fpsyt.2019.00385. 10.3389/fpsyt.2019.00385 PMC6555089 31214062

[3] Bateman AW, Fonagy P (2006). Mentalization-based treatment for borderline personality disorder: a practical guide.

[4] Beck, E., Bo, S., Jorgensen, M.S., Gondan, M., Poulsen, S., Storebo, O.J. et al. (2019). Mentalization‐based treatment in groups for adolescents with. borderline personality disorder: a randomized controlled trial. J. Child Psycholology and Psychiatry. 10.1111/jcpp.13152.

[5] Becker DF, Grilo CM, Edell WS, McGlashan TH (2002). Diagnostic efficiency of borderline personality disorder criteria in hospitalized adolescents: comparison with hospitalized adults. Am J Psychiatry.

[6] Bender DS, Dolan RT, Skodol AE, Sanislow CA, Dyck IR, McGlashan TH, Gunderson JG (2001). Treatment utilization by patients with personality disorders. Am J Psychiatry.

[7] Birchwood M, Lester H, McCarthy L, Jones P, Fowler D, Amos T (2014). The UK national evaluation of the development and impact of early intervention services (the National EDEN studies): study rationale, design and baseline characteristics. Early Interv Psychiatry.

[8] Bleiberg E (2001). Treating personality disorders in children and adolescents: a relational approach.

[9] Chanen AM, Berk M, Thompson K (2016). Integrating early intervention for borderline personality disorder and mood disorders. Harv Rev Psychiatry.

[10] Chanen AM, Jackson HJ, McCutcheon LK, Jovev M, Dudgeon P, Yuen HP (2008). Early intervention for adolescents with borderline personality disorder using cognitive analytic therapy: randomised controlled trial. Br J Psychiatry.

[11] Chanen AM, McCutcheon LK, Germano D, Nistico H, Jackson HJ, McGorry PD (2009). The HYPE clinic: an early intervention service for borderline personality disorder. J Psychiatr Pract.

[12] Chanen AM, Sharp CS, Hoffman P, Global Alliance for Prevention and Early Intervention for Borderline Personality Disorder (2017). Prevention and early intervention for borderline personality disorder: a novel public health priority. World Psychiatry.

[13] Cohen P, Crawford TN, Johnson JG, Kasen S (2005). The children in the community study of the developmental course of personality disorder. J Personal Disord.

[14] Debast I, Rossi G, Feenstra DF, Hutsebaut J (2017). Developmentally sensitive markers of personality functioning in adolescents: age-specific and age-neutral expressions. Personal Disord Theory Res Treat.

[15] De Clercq B, van Leeuwen K, Van den Noortgate W, De Bolle M, De Fruyt F (2009). Childhood personality pathology: dimensional stability and change. Dev Psychopathol.

[16] De Fruyt F, De Clercq B (2014). Antecedents of personality disorder in childhood and adolescence: toward an integrative developmental model. Annu Rev Clin Psychol.

[17] Dumontheil I, Apperly IAI, Blakemore S-J (2010). Online usage of theory of mind continues to develop in late adolescence. Dev Sci.

[18] First MB, Spitzer RL, Gibbon M, Williams JBW (1997). Structured clinical interview for DSM-IV Axis I disorders.

[19] First MB, Spitzer RL, Gibbon M, Williams JBW, Benjamin L (1996). Structured clinical interview for DSM-IV Axis II personality disorders (SCID-II).

[20] Fok MLY, Hayes RD, Chang CK, Stewart R, Callard FJ, Moran P (2012). Life expectancy at birth and all-cause mortality among people with personality disorder. J Psychosom Res.

[21] Fonagy P, Allison E (2014). The role of mentalizing and epistemic trust in the therapeutic relationship. Psychotherapy.

[22] Fonagy P, Gergely G, Jurist EL, Target M (2002). Affect regulation, mentalization, and the development of the self.

[23] Fonagy P, Luyten P, Allison E (2015). Epistemic petrification and the restoration of epistemic trust: a new conceptualization of borderline personality disorder and its psychosocial treatment. J Personal Disord.

[24] Fonagy P, Luyten P, Allison E, Campbell C (2017). What we have changed our minds about: part 1. Borderline personality disorder as a limitation of resilience. Borderline Personal Disord Emot Dysregul.

[25] Fonagy P, Luyten P, Allison E, Campbell C (2017). What we have changed our minds about: part 2. Borderline personality disorder, epistemic trust and the developmental significance of social communication. Borderline Personal Disord Emot Dysregul.

[26] Grant BF, Chou SP, Goldstein RB, Boji-Huang MPH, Stinson FS, Saha TD (2008). Prevalence, correlates, disability, and comorbidity of DSM-IV borderline personality disorder: results from the wave 2 national epidemiologic survey on alcohol and related conditions. J Clin Psychiatry.

[27] Ha C, Balderas JC, Zanarini MC, Oldham J, Sharp C (2017). Psychiatric comorbidity in hospitalized adolescents with borderline personality disorder. J Clin Psychiatry.

[28] Hillebrandt H, Dumontheil I, Blakemore S-J, Roiser JP (2013). Dynamic causal modelling of effective connectivity during perspective taking in a communicative task. Neuroimage.

[29] Hutsebaut J, Videler AC, Verheul R, van Alphen SPJ. Managing borderline personality disorder from a life course perspective: clinical staging and health management. Personal Disord. 2019; In press. 10.1037/per0000341 31144839

[30] Johnson JG, Cohen P, Kasen S, Skodol AE, Hamagami F, Brooks JS (2000). Age-related change in personality disorder trait levels between early adolescence and adulthood: a community-based longitudinal investigation. Acta Psychiatr Scand.

[31] Johnson JG, First MB, Cohen P, Skodol AE, Kasen S, Brook JS (2005). Adverse outcomes associated with personality disorder not otherwise specified in a community sample. Am J Psychiatr.

[32] Kasen S, Cohen P, Skodol AE, First MB, Johnson JG, Brooks JS, Oldham JM (2007). Comorbid personality disorder and treatment use in a community sample of youths: a 20-year follow-up. Acta Psychiatr Scand.

[33] Laurenssen EMP, Feenstra DJ, Busschbach JJV, Hutsebaut J, Bales DL, Luyten P, Verheul R, Noom MJ. Feasibility of mentalization-based treatment for adolescents with borderline symptoms: a pilot study. Psychother Theory Res Pract Train. 2013b;51(1). 10.1037/a0033513. 10.1037/a0033513 24059741

[34] Laurenssen EMP, Hutsebaut J, Feenstra DJ, van Busschbach JJV, Luyten P (2013). Diagnosis of personality disorders in adolescents: a study among psychologists. Child Adolesc Psychiatry Ment Health.

[35] Linehan MM (1993). Cognitive-behavioral treatment of borderline personality disorder.

[36] Moran P, Stewart R, Brugha T, Bebbington P, Bhugra D, Jenkins R, Coid JW (2007). Personality disorder and cardiovascular disease: results from a national household survey. J Clin Psychiatry.

[37] Mutlu AK, Schneider M, Debbané M, Badoud D, Eliez S, Schaer M (2013). Sex differences in thickness, and folding developments throughout the cortex. NeuroImage.

[38] Penner F, Wall K, Jardin C, Brown JL, Sales JM, Sharp C (2019). A study of risky sexual behavior, beliefs about sexual behavior, and sexual self-efficacy in adolescent inpatients with and without borderline personality disorder. Personal Disord Theory Res Treat.

[39] Poznyak E, Morosan L, Perroud N, Speranza M, Badoud D, Debbané M (2019). Roles of age, gender and psychological difficulties in adolescent mentalizing. J Adolesc.

[40] Rossouw, T.I., & Fonagy, P. (2012). Mentalization-based treatment for self-harm in adolescents: a randomized controlled trial. Journal of the American Academy of Child and Adolescent Psychiatry, 51, 1304–1313. 10.1016/j.jaac.2012.09.018 23200287

[41] Scott J, Leboyer M, Hickie I, Berk M, Kapczinski F, Frank E (2013). Clinical staging in psychiatry: a cross-cutting model of diagnosis with heuristic and practical value. Br J Psychiatry.

[42] Scalzo F, Hulbert CA, Betts JK, Cotton SM, Chanen AM. Substance use in youth with borderline personality disorder. J Personal Disord. 2017;31:315–331.

[43] Sharp C, Wall K (2018). Personality pathology grows up: adolescence as a sensitive period. Curr Opin Psychol.

[44] Sharp C, DeClercq B. Personality pathology in children and adolescents. In: Gratz K, Lejeuz C, editors. Handbook of personality disorders. Cambridge: Cambridge University Press; 2020.

[45] Sharp C, Ha C, Carbone C, Kim S, Perry K, Williams L, Fonagy P (2013). Hypermentalizing in adolescent inpatients: treatment effects and association with borderline traits. J Personal Disord.

[46] Sharp C, Steinberg L, Michonski J, Kalpakci A, Fowler C, Frueh C, Fonagy P (2019). DSM borderline criterion function across age groups: a cross-sectional mixed-method study. Assessment.

[47] Sharp C, Wright AGC, Fowler JC, Frueh BC, Allen JG, Oldham J, Clark L (2015). The structure of personality pathology: both general (‘g’) and specific (‘s’) factors?. J Abnorm Psychol.

[48] Skodol AE, Clark LA, Bender DS, Krueger RF, Morey LC, Verheul R, Alarcon RD, Bell CC, Siever LJ, Oldham JM (2011). Proposed changes in personality and personality disorder assessment and diagnosis for DSM-5. Part I description and rationale. J Pers Disord.

[49] Stepp S, Lazarus SA, Byrd AL (2016). A systematic review of risk factors prospectively associated with borderline personality disorder: taking stock and moving forward. J Personal Disord.

[50] Stepp SD, Pilkonis PA, Hipwell AE, Loeber R, Stouthamer-Loeber M (2010). Stability of borderline personality disorder features in girls. J Personal Disord.

[51] Thompson KN, Betts J, Jovev M, Nyathi Y, McDougall E, Chanen AM (2019). Sexuality and sexual health among female youth with borderline personality disorder pathology. Early Interv Psychiatry.

[52] Videler AC, Hutsebaut J, Schulkens JEM, Sobczak S, van Alphen SPJ (2019). A life span perspective on borderline personality disorders. Curr Psychiatry Opin.

[53] Westen D, Shedler J, Durett C, Glass S, Martens A (2003). Personality diagnoses in adolescence: DSM-IV axis II diagnoses and an empirically derived alternative. Am J Psychiatr.

[54] Winsper C, Marwaha S, Lereya ST, et al. (2015) Clinical and psychosocial outcomes of borderline personality disorder in childhood and adolescence: A systematic review. Psychological Medicine 45: 2237–2251. 10.1017/S0033291715000318 25800970

[55] Winsper C, Wolke D, Scott J, Sharp C, Thompson A, Marwaha S. Psychopathological outcomes of adolescent borderline personality disorder symptoms. Aust N Z J Psychiatry. 2019. 10.1177/0004867419882494. 10.1177/0004867419882494 31647321

[56] Wright AGC, Zalewski M, Hallquist MN, Hipwell AE, Stepp SD (2016). Developmental trajectories of borderline personality disorder symptoms and psychosocial functioning in adolescence. J Personal Disord.

[57] Zanarini MC, Conkey LC, Temes CM, Fitzmaurice GM. Randomized controlled trial of web-based psychoeducation for women with borderline personality disorder. J Clin Psychiatry. 2018;79(3). 10.4088/JCP.16m11153. 10.4088/JCP.16m11153 PMC5764827 28703950

[58] Zanarini MC, Frankenburg FR, Reich DB, Fitzmaurice G (2012). Attainment and stability of sustained symptomatic remission and recovery among borderline patients and Axis II comparison subjects: a 16-year prospective follow-up study. Am J Psychiatr.

[59] Zanarini MC, Temes CM, Magni LR, Fitzmaurice GM, Aguirre BA, Goodman M (2017). Prevalence rates of borderline symptoms reported by adolescent inpatients with BPD, psychiatrically healthy adolescents and adult inpatients with BPD: BPD symptoms in adolescence. Personal Ment Health.

[60] Zanarini MC, Vujanovic AA, Parachini EA, Boulanger JL, Frankenburg FR, Hennen J (2003). A screening measure for BPD: the McLean screening instrument for borderline personality disorder (MSI-BPD). J Pers Disord.

